# Gestational weight gain mediates the effects of energy intake on birth weight among singleton pregnancies in the Japan Environment and Children’s Study

**DOI:** 10.1186/s12884-022-04898-3

**Published:** 2022-07-16

**Authors:** Marina Minami, Naw Awn J-P, Shuhei Noguchi, Masamitsu Eitoku, Sifa Marie Joelle Muchanga, Naomi Mitsuda, Kaori Komori, Kahoko Yasumitsu-Lovell, Nagamasa Maeda, Mikiya Fujieda, Narufumi Suganuma, the Japan Environment and Children’s Study (JECS) Group, Michihiro Kamijima, Shin Yamazaki, Yukihiro Ohya, Reiko Kishi, Nobuo Yaegashi, Koichi Hashimoto, Chisato Mori, Shuichi Ito, Zentaro Yamagata, Hidekuni Inadera, Takeo Nakayama, Hiroyasu Iso, Masayuki Shima, Youichi Kurozawa, Narufumi Suganuma, Koichi Kusuhara, Takahiko Katoh

**Affiliations:** 1 Department of Environmental Medicine, Kochi Medical School, Kochi University, Nankoku, Kochi 783-8505 Japan; 2 Department of Obstetrics and Gynecology, Kochi Medical School, Kochi University, Nankoku, Kochi Japan; 3 Department of Pediatrics, Kochi Medical School, Kochi University, Nankoku, Kochi Japan

**Keywords:** Birth weight, Energy intake, Fetal development, Gestational weight gain, Pregnancy, Small for gestational age

## Abstract

**Background:**

Extra energy intake is commonly recommended for pregnant women to support fetal growth. However, relevant data regarding variations in energy intake and expenditure, body mass index and gestational weight gain (GWG) are frequently not considered. This study aimed to investigate how energy intake during pregnancy and gestational weight gain (GWG) are associated with birth weight.

**Methods:**

Early pregnant women were recruited into a Japanese nationwide prospective birth cohort study between 2011 and 2014. We analysed data of 89,817 mother-child pairs of live-born non-anomalous singletons after excluding births before 28 weeks or after 42 weeks. Energy intake during pregnancy was estimated from self-administered food frequency questionnaires (FFQ) and was stratified into low, medium, and high. Participants completed the FFQ in mid-pregnancy (mean 27.9 weeks) by recalling food consumption at the beginning of pregnancy. Effects of energy intake on birth weight and mediation by GWG were estimated using the Karlson–Holm–Breen method; the method separates the impact of confounding in the comparison of conditional and unconditional parameter estimates in nonlinear probability models. Relative risks and risk differences for abnormal birth size were calculated.

**Results:**

Mean daily energy intake, GWG, and birth weight were 1682.1 (533.6) kcal, 10.3 (4.0) kg, and 3032.3 (401.4) g, respectively. 6767 and 9010 women had small-for-gestational-age and large-for-gestational-age infants, respectively. Relative to low energy intake, moderate and high intakes increased adjusted birth weights by 13 g and 24 g, respectively: 58 and 69% of these effects, respectively, were mediated by GWG. Compared with the moderate energy intake group, the low energy intake group had seven more women per 1000 women with a small-for-gestational-age birth, whereas the high energy intake group had eight more women per 1000 women with a large-for-gestational-age birth.

**Conclusion:**

GWG mediates the effect of energy intake on birth weight. All pregnant women should be given adequate nutritional guidance for optimal GWG and fetal growth.

## Background

Fetal growth and birth weight are determined by a complex interaction among several maternal, pregnancy-related, and fetal-related factors. Extreme pre-pregnancy body mass indexes (BMIs), insufficient or excessive gestational weight gain (GWG), imbalanced nutrition, alcohol consumption and smoking, maternal diabetes and hypertension, multiple pregnancies, and preterm birth have all been associated with abnormal size of newborns [1–5].

Appropriate GWG has been investigated based on pre-pregnancy BMI, and several organisations have proposed GWG guidelines [6, 7] for pregnant women to lower their risk of giving birth to newborns of abnormal size or birth weight. Similarly, the energy requirements for pregnant women have been investigated based on their pre-pregnancy BMI and stage of pregnancy [8, 9]. Findings indicated that additional energy is needed during pregnancy to support fetal growth, the incremental increases in the sizes of the maternal tissues, and changes in maternal energy metabolism. Thus, recommendations have been published [10, 11] for the intake of extra energy to meet the increased demand, depending on pre-pregnancy body weight and the intensity of physical activity. However, the relationship between maternal energy intake and fetal growth is unclear. Some investigators [12–14] found no associations between energy intake and fetal growth and birth weight. However, they do document positive associations between GWG and maternal energy intake and birth weight. Awareness of gaining weight may influence the food consumption of a pregnant woman and hence the newborn birth weight [15]; however, it is more likely that energy consumed during pregnancy is deposited in the maternal and fetal tissues and perceived as GWG [8, 9]. Thus, it is plausible that the effects of energy intake on fetal growth and birth weight may be mediated by GWG, which has not been investigated previously.

Therefore, the present study aimed to investigate how energy intake during pregnancy and GWG are associated with birth weight and distinguish between the direct association of energy intake and indirect association mediated by GWG. Furthermore, we aimed to evaluate how different levels of energy intake affect birth size (determined by weight for gestational age at birth) in a cohort of singleton pregnancies from the Japan Environment and Children’s Study (JECS) [16].

## Methods

### Study participants

The JECS aims to explore environmental factors that affect the health and development of children and is being by the Environment, Japan. The detailed methodology and baseline profile of the study participants have been reported previously [16, 17]. Briefly, approximately 100,000 early pregnant women who live in 15 designated Study Areas across Japan were recruited between January 2011 and March 2014. They will be followed until the participating children reach 13 years of age. Participants were considered eligible if they met the following criteria: (1) they resided in a Study Area at the time of recruitment and were expected to reside continually in Japan, (2) they were expected to give birth after August 1, 2011, and (3) they were capable of understanding the Japanese language and completing a self-administered questionnaire.

**Fig. 1:**
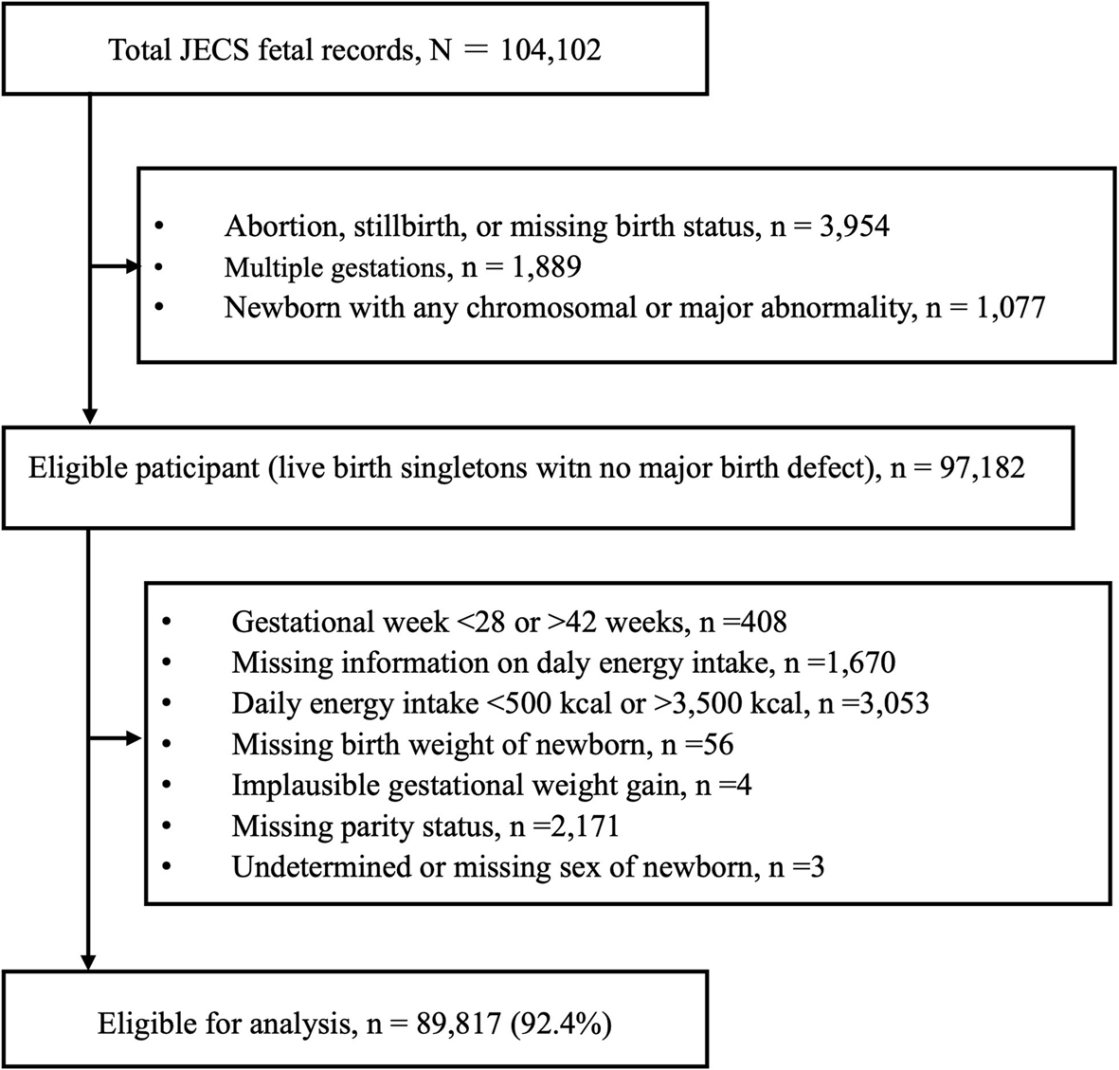
Participant flowchart. JECS, Japan Environment and Children’s Study

This study uses the jecs-ag-20,160,424 dataset (released in June 2016) from the JECS, which comprises information from 104,102 fetal records. Therefore, formal sample size calculation was not performed in the present study. We restricted this study to 97,182 pregnancies resulting in live singleton births with no major birth defects. Then, we excluded participants based on predetermined exclusion criteria related to our exposure and outcome variables (Fig. 1): births before 28 weeks or after 42 weeks; missing or implausible daily energy intake (we used commonly applied values of < 500 or > 3500 kcal/day to define implausible daily energy intake [18, 19]); missing birth weight; implausible data on GWG (i.e. body weight recorded instead of GWG); and missing parity or newborn sex.

### Exposure and outcome assessment

Nutritional information during pregnancy was obtained via a validated food frequency questionnaire (FFQ) [20, 21]. The FFQ was administered twice during pregnancy. The first time was in the first trimester to investigate the pre-pregnancy food frequency. The second FFQ conducted in mid-pregnancy (after week 22) was used in this study. The participants completed the FFQ by recalling their food consumption at the beginning of pregnancy (i.e., the point at which they realised that they were pregnant) and recording the quantity of each food item and the frequency with which it was consumed. Frequency categories ranged from ‘almost never’ to ‘7 or more times per day’ (or ‘10 glasses per day’ for beverages). Daily total energy intake was estimated using a food composition table that was developed based on the Standardised Tables of Food Composition in Japan (2010 edition) [22].

Anthropometric measurements of newborns, including birth weight (in grams) and gestational age at birth (in weeks), were extracted from medical records transcripts. Gestational age was calculated from the first day of the last menstrual period or by using ultrasound examinations of the fetus during the first trimester. Birth size was defined as small-for-gestational-age (SGA), appropriate-for-gestational-age (AGA), and large-for-gestational-age (LGA) for birth weights below the 10th percentile, 10th to 90th percentile, or above the 90th percentile, respectively, according to the Japanese parity and sex-specific neonatal birth weight chart for gestational age at birth [23].

### Mediator and other covariates

Demographic, medical condition, and pregnancy-related data were collected through self-administered standard questionnaires or from medical records. GWG (mediator variable) was calculated by subtracting the self-reported pre-pregnancy body weight from the last measured weight closest to childbirth. Pre-pregnancy BMI was calculated by dividing the self-reported pre-pregnancy body weight (in kilograms) by the square of the height (in metres). The following information was also obtained: socioeconomic and lifestyle information, such as education, alcohol consumption, and smoking; occupation during pregnancy; physical activity levels (metabolic equivalent of a task measured as minutes per day) [24]; medical history of chronic diseases (diabetes, hypertension, heart disease, and kidney disease); nausea and vomiting during pregnancy; and receipt of health guidance.

### Statistical analysis

Daily energy intake during pregnancy was stratified into three levels (low, moderate, and high) comprising approximately equal numbers of participants. The tertiles were the 33.4 and 67.9 percentiles, corresponding to the energy intake values of 1412 kcal and 1856 kcal, respectively. GWG was analysed as a continuous variable. Birth weight was studied as a continuous variable and birth size in categories (SGA, AGA, and LGA). Pre-pregnancy BMI was stratified as ‘underweight’ (< 18.5 kg/m^2^), ‘normal weight’ (18.5–24.9 kg/m^2^), and ‘overweight and obese’ (≥25 kg/m^2^). Because the Japanese physique is small and underweight by global standards, the Ministry of Health, Labour and Welfare (MHLW) has provided a guideline for pregnancy weight gain, calculated from the Japanese average and by pre-pregnancy BMI category. The participants were grouped into ‘low’, ‘appropriate’, and ‘high’ GWG categories (for descriptive statistics) within each pre-pregnancy BMI stratum. GWG was stratified by applying the appropriate GWG ranges of 9–12 kg and 7–12 kg for underweight and normal-weight pregnant women, respectively, according to the recommendations of the Ministry of Health, Labour, and Welfare, Japan [7]. Since there were no published recommendations for the overweight group, we used a GWG range of 5–7 kg, adopted from the Japanese Society for the Study of Obesity guidelines [25]. GWGs of ≤7 kg and ≤ 5 kg were applied for overweight (25–29.9 kg/m^2^) and obese (≥30 kg/m^2^) pregnant women, respectively. The participants were categorised into three age groups: ≤25, 26–35, and ≥ 36 years. Maternal educational attainment was stratified into three groups: ‘high school or less’, ‘vocational school/college’, and ‘university or higher education’. Considering that activity level may vary across job types [26], occupation during pregnancy was categorised as ‘unemployed’ (including students and women not classifiable by occupation), ‘low physical job’ (e.g. managers, engineers, and clerks), ‘moderate physical job’ (e.g. sales, services, and security workers), and ‘high physical job’ (e.g. manufacturing process workers, farmers, construction workers, carrying workers, and cleaners). Physical activity during pregnancy was grouped into three levels (low, moderate, and high) with approximately equal numbers of participants in each group. Other variables, such as alcohol consumption and smoking during pregnancy, chronic diseases, nausea and vomiting during pregnancy, and receipt of health guidance, were dichotomised as present (yes) or absent (no). Non-smoking and non-drinking women also included those who quit before or after they realised that they were pregnant.

**Fig. 2:**
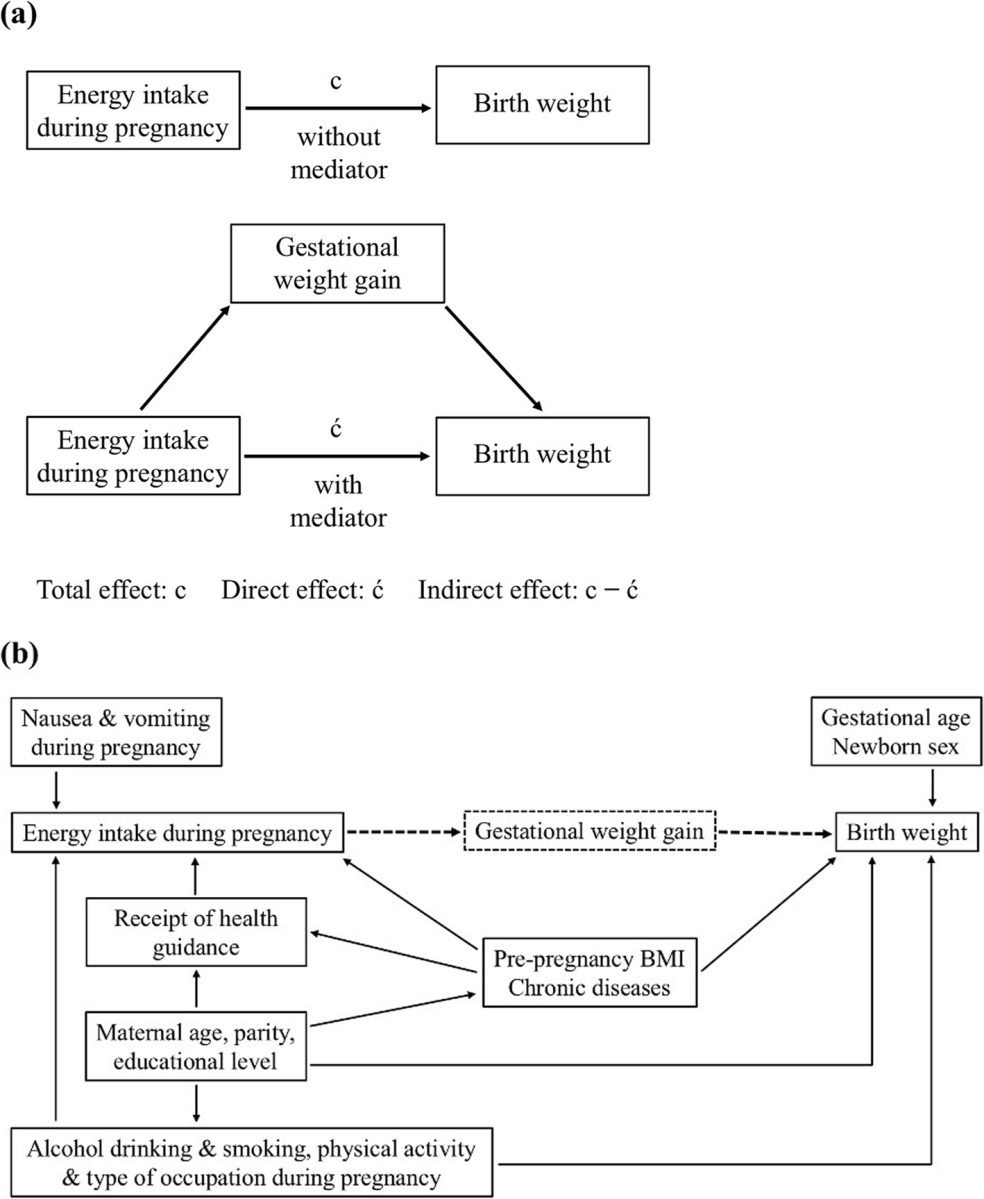
Schematic illustration of the conceptual framework. **a** Direct and indirect effects of energy intake during pregnancy on birth weight. **b** Directed acyclic graph for the association between energy intake during pregnancy and birth weight. The dotted lines represent the research question and the solid lines the covariates adjusted for

Descriptive statistics of the participants were calculated for the entire study population and according to the levels of energy intake. The mean and standard deviation for continuous variables and numbers and percentages for categorical variables are reported. Since our primary aim was to examine how energy intake during pregnancy and GWG are associated with birth weight, we performed a mediation analysis. Figure 2 illustrates the conceptual framework of our study. We assumed energy intake preceded GWG in our analyses. The following points were the reasons for this assumption. First, the participants completed the FFQ by recalling their food consumption from conception (although FFQ was administered during mid-pregnancy). Second, the rate of weight gain is slower in the first trimester compared to the second or third [27] (although we used the total GWG). We compared the coefficients of the exposure (energy intake during pregnancy) derived from models with and without the mediator (GWG) (Fig. 2a) by employing the Karlson–Holm–Breen method using a linear regression model [28]. The Karlson–Holm–Breen method allowed, in a single command, the separation of the total effect of energy intake (c) into direct (ć) and indirect effects mediated by GWG (c – ć) while controlling for potential confounding variables. Based on the literature [1–5, 29, 30] and considering the causal relationship with exposure and outcome variables (Fig. 2b), the following factors were considered potential confounders and controlled for in the mediation analysis: maternal age (years), parity, educational level, pre-pregnancy BMI (kg/m^2^), type of occupation and physical activity level during pregnancy, alcohol consumption and smoking during pregnancy, the presence of chronic diseases, receipt of health guidance, and nausea and vomiting during pregnancy. To increase the precision of the estimates, newborn sex and gestational age (weeks) were also adjusted. Next, we evaluated the associations between levels of energy intake (moderate energy intake as the referent) and birth size using a multinomial logistic regression model. The crude and adjusted relative risk ratios (RRR), adjusted risk differences, and corresponding 95% confidence intervals (CIs) for SGA and LGA births were reported. The same factors included in the mediation analysis, except newborn sex and gestational age, were included in the adjusted model. Additionally, we examined the characteristics of the women according to their inclusion status in our analysis. All statistical analyses were performed using Stata/MP version 15.1 (StataCorp., College Station, TX, USA). Statistical significance was defined as a two-sided *P*-value < 0.05.

### Ethics statement

This study was conducted according to the principles expressed in the Declaration of Helsinki. All procedures involving human participants were approved by the Japan Ministry of the Environment’s Institutional Review Board on Epidemiological Studies (No. 100910001) and the Ethics Committees of all participating institutions. Written informed consent was obtained from all participants.

## Results

Of the 97,182 eligible pregnancies reviewed, 7365 were excluded because they met the predetermined exclusion criteria. Thus, 89,817 (92.4%) mother-child pairs of live-born non-anomalous singletons remained for analyses (Fig. 1). The mean (standard deviation) that participants completed FFQ was 27.9 (6.5) weeks. The mean birth weight in the low, moderate, and high energy intake groups and in the entire study population were 3016 (402.9) g, 3034.7 (399.6) g, 3046. 4(401.1) g, and 3032.3 (401.4) g, respectively. Of the 89,817 women assessed, 6767 (7.5%) and 9010 (10.0%) gave birth to SGA and LGA infants, respectively. The proportions of SGA births were 8.1, 7.4, and 7.2%, and those of LGA births were 9.7, 9.8, and 10.6%, respectively, in the low, moderate, and high energy intake groups.

Table 1 reports descriptive statistics of participants according to the levels of energy intake. Younger, nulliparous women who did not exceed high school, and women who had extreme pre-pregnancy BMIs, reported less activity, and gained less weight during pregnancy were more prevalent in the low energy intake group than in the other groups. In contrast, parous women who reported being active during pregnancy and gained greater weight were more frequent in the high energy intake group. Table 2 presents the estimated linear regression coefficients for the association between energy intake during pregnancy and birth weight and the portion of the associations mediated by GWG. It shows that, relative to the low energy intake group, mean birth weights were increased by 13.43 g and 23.76 g in the moderate and high energy intake groups, respectively. The results also show that GWG mediated a significant portion of the effect of energy intake on birth weight; the proportion mediated by GWG was 58.4 and 68.5% in the moderate and high energy intake groups, respectively. Table 3 presents relative risk ratios and risk differences for SGA and LGA births. Compared with the moderate energy intake group, the risk of SGA birth was significantly higher in the low energy intake group (adjusted RRR: 1.12, 95% CI: 1.04–1.19), whereas the risk of LGA birth was significantly higher in the high energy intake group (adjusted RRR: 1.09, 95% CI: 1.03–1.16). It also shows that, compared with the moderate energy intake group, the low energy intake group had seven more women per 1000 women with an SGA birth, whereas the high energy intake group had eight more women per 1000 women with an LGA birth. Table 4 shows the characteristics of women according to their inclusion status in the analyses. The included and excluded groups largely resembled each other; however, the excluded group had a higher proportion of younger women, those who did not exceed high school, and those who engaged in a job that required moderate physical activity. A higher proportion of the excluded women gained more weight than recommended but had a higher proportion of SGA births than that for the included women.

**Table 1:** Participants’ characteristics according to level of energy intake during pregnancy

	Energy intake level^**a**^			
Participant (Number)	All (89,817)	Low (30,035)	Moderate (30,914)	High (28,868)
	**Mean (SD)**			
Energy intake, kcal/d	1682.1(536.6)	1144.9(200.2)	1623.3(126.5)	2304.1(382.5)
Maternal age, year	31.2(5.0)	30.6(5.2)	31.4(4.9)	31.6(4.9)
Pre-pregnancy BMI, kg/m^2^	21.2(3.3)	21.3(3.4)	21.2(3.2)	21.3(3.3)
GWG, kg	10.3(4.0)	10.0(4.1)	10.3(3.9)	10.6(4.0)
Gestational age, weeks	38.9(1.4)	38.9(1.4)	38.9(1.4)	38.8(1.4)
Birth weigh	3032.3(401.4)	3016.1(402.9)	3034.7(399.6)	3046.4(401.1)
	**Number (%)**			
Maternal age				
Up to 25 years	11,952(13.3)	5103(17.0)	3650(11.8)	3199(11.1)
26–35 years	58,970(65.7)	19,237(64.1)	20,643(66.8)	19,090(66.1)
36 or more years	18,892(21.0)	5694(19.0)	6619(21.4)	6579(22.8)
Missing	3	1	2	0
Pre-pregnancy BMI, kg/m^2^				
Underweight, < 18.5	14,451(16.1)	5006(16.7)	5006(16.2)	4439(15.4)
Normal weight, 18.5–24.9	65,772(73.2)	21,609(72.0)	22,829(73.9)	21,334(73.9)
Overweight and obese, ≥25	9551(10.6)	3400(11.3)	3074(9.9)	3077(10.7)
Missing	43(0.1)	20(0.1)	5(0.0)	18(0.1)
GWG^b^				
Low	15,917(17.7)	5942(19.8)	5471(17.7)	4504(15.6)
Appropriate	41,932(46.7)	13,958(46.5)	14,631(47.3)	13,343(46.2)
High	30,238(33.7)	9566(31.9)	10,162(32.9)	10,510(36.4)
Missing	1730(1.9)	569(1.9)	650(2.1)	511(1.8)
Parity				
Nullipara	36,240(40.4)	14,055(46.8)	12,334(39.9)	9851(34.1)
Multipara	53,577(59.7)	15,980(53.2)	18,580(60.1)	19,017(65.9)
Educational level				
High school or less	32,419(36.1)	12,225(40.7)	10,396(33.6)	9798(33.9)
Vocational school/College	37,680(42.0)	12,015(40.0)	13,099(42.4)	12,566(43.5)
University or higher	19,395(21.6)	5668(18.9)	7311(23.7)	6416(22.2)
Missing	323(0.4)	127(0.4)	108(0.4)	88(0.3)
Occupation during pregnancy^c^				
Non-employed	29,422(32.8)	9461(31.5)	10,290(33.3)	9671(33.5)
Low physical job	33,884(37.7)	11,021(36.7)	12,065(39.0)	10,798(37.4)
Moderate physical job	18,254(20.3)	6608(22.0)	5855(18.9)	5791(20.1)
High physical job	3971(4.4)	1500(5.0)	1313(4.3)	1158(4.0)
Missing	4286(4.8)	1445(4.8)	1391(4.5)	1450(5.0)
Physical activity level (MET-min/d)				
Low	30,351(33.8)	10,704(35.6)	10,507(34.0)	9140(31.7)
Moderate	27,981(31.2)	9362(31.2)	9970(32.3)	8649(30.0)
High	27,312(30.4)	8547(28.5)	9089(29.4)	9676(33.5)
Missing	4173(4.7)	1422(4.7)	1348(4.4)	1403(4.9)
Smoking during pregnancy				
Yes	4052(4.5)	1549(5.2)	1184(3.8)	1319(4.6)
No	85,049(94.7)	28,217(94.0)	29,508(95.5)	27,324(94.7)
Missing	716(0.8)	269(0.9)	222(0.7)	225(0.8)
Drinking alcohol during pregnancy				
Yes	2521(2.8)	698(2.3)	875(2.8)	948(3.3)
No	86,679(96.5)	29,100(96.9)	29,852(96.6)	27,727(96.1)
Missing	617(0.7)	237(0.8)	187(0.6)	193(0.7)
Nausea and vomiting				
Yes	74,316(82.7)	24,778(82.5)	25,688(83.1)	23,850(82.6)
No	15,200(16.9)	5144(17.1)	5125(16.6)	4931(17.1)
Missing	301(0.3)	113(0.4)	101(0.3)	87(0.3)
Chronic diseases^d^				
Yes^c^	2453(2.7)	843(2.8)	857(2.8)	753(2.6)
No	85,858(95.6)	28,710(95.6)	29,476(95.4)	27,672(95.9)
Missing	1506(1.7)	482(1.6)	581(1.9)	443(1.5)
Receipt of health guidance				
Yes	9528(10.6)	3307(11.0)	3236(10.5)	2985(10.3)
No	9528(10.6)	3307(11.0)	3236(10.5)	2985(10.3)
Missing	1626(1.8)	510(1.7)	610(2.0)	506(1.8)

*BMI* body mass index, *GWG* gestational weight gain, *MET* metabolic equivalent, *SD* standard deviation

^a^Energy intake level was categorised as low, moderate, or high, with approximately equal numbers of participants in each group. The tertiles were the 33.4 and 67.9 percentiles, corresponding to 1412 kcal and 1856 kcal, respectively

^b^GWG was stratified by applying the appropriate GWG ranges of 9 to12 kg, 7 to 12 kg, and 5 to 7 kg for underweight, normal-weight, and overweight and obese pregnant women, respectively

^c^Categorised as ‘unemployed’ (including students and women not classifiable by occupation), ‘low physical job’ (e.g. managers, engineers, and clerks), ‘moderate physical job’ (e.g. sales, services, and security workers), and ‘high physical job’ (e.g. manufacturing process workers, farmers, construction workers, carrying workers, and cleaners)

^d^diabetes, hypertension, heart disease, or kidney disease

**Table 2:** Estimated linear regression coefficients for the associations between energy intake during pregnancy and birth weight, and for the effects mediated by GWG

	Birth weight	
	**Coefficients**^**a**^	**95% CI**
**Energy intake level**^**b**^		
***Low***	Ref.	
***Moderate***		
Total effect	13.43	7.99–18.86
Direct effect	5.59	0.15–11.02
Indirect effect	7.84	5.92–9.76
Proportion mediated by GWG	58.4%	
***High***		
Total effect	23.76	18.20–29.32
Direct effect	7.50	1.92–13.08
Indirect effect	16.27	14.31–18.23
Proportion mediated by GWG	68.5%	

*CI* confidence interval, *GWG* gestational weight gain

Mediation analysis was performed by employing the Karlson–Holm–Breen method using a linear regression model. The model was adjusted for maternal age, parity, pre-pregnancy body mass index, educational level, type of occupation during pregnancy, physical activity level during pregnancy, alcohol consumption and smoking during pregnancy, nausea and vomiting during pregnancy, presence of chronic disease, receipt of health guidance, female sex, and gestational age (weeks)

^a^Regression coefficients can be interpreted as, for example, mean birth weight was increased by 13.43 g when moving from low to moderate energy intake

^b^Energy intake was stratified into three levels (low, moderate, or high) comprising approximately equal numbers of participants. The tertiles were the 33.4 and 67.9 percentiles, corresponding to 1412 kcal and 1856 kcal, respectively

**Table 3:** Relative risk ratios (RRR) and risk differences (RD) for abnormal birth sizes according to the level of energy intake during pregnancy

	Cases, n (%)	RRR	95% CI	RRR^**a**^	95% CI	RD^**a,b**^	95% CI
**Small-for-gestational age**							
Energy intake level							
Low	2428 (8.1)	1.11	1.04–1.18	1.12	1.04–1.19	0.007	0.003–0.012
Moderate	2272(7.4)	Ref.		Ref.		Ref.	
High	2064(7.2)	0.98	0.92–1.04	1.00	0.93–1.07	−0.001	−0.005–0.004
**Large-for-gestational age**							
Energy intake level							
Low	2908(9.7)	0.99	0.94–1.05	0.97	0.91–1.02	−0.004	− 0.009–0.001
Moderate	3041(9.8)	Ref.		Ref.		Ref.	
High	3061(10.6)	1.09	1.03–1.14	1.09	1.03–1.16	0.008	0.003–0.013

*CI* confidence interval

Multinomial logistic regression models were used to estimate the relative risk ratios and risk differences

^a^Adjusted for maternal age, parity, pre-pregnancy body mass index, educational level, type of occupation during pregnancy, physical activity level during pregnancy, alcohol consumption and smoking during pregnancy, nausea and vomiting during pregnancy, presence of chronic disease, and receipt of health guidance

^b^Risk difference can be interpreted as, for example, when compared to moderate energy intake, low energy intake had 7 more women per 1000 women with an SGA birth

**Table 4:** Participants’ characteristics according to inclusion status in the current study

	Eligible	Included	Excluded
**Total, Number (%)**	97,182	89,817(92.4)	7365(7.6)
	**Mean (SD)**		
Maternal age, year	31.1(5.1)	31.2(5.0)	30.3(5.4)
Pre-pregnancy BMI, kg/m^2^	21.2(3.3)	21.2(3.3)	21.3(3.4)
GWG, kg	10.3(4.9)	10.3(4.0)	10.7(11.2)
Birth weight, g	3026.3(415.2)	3032.3(401.4)	2953.2(553.2)
	**Number**^**a**^ **(%)**		
Maternal age			
Up to 25 years	13,416(13.8)	11,952(13.3)	1464(19.9)
26–35 years	63,497(65.3)	58,970(65.7)	4527(61.5)
36 or more years	20,264(20.9)	18,892(21.0)	1372(18.6)
Pre-pregnancy BMI, kg/m^2^			
Underweight, < 18.5	15,680(16.2)	14,451(16.1)	1229(16.9)
Normal weight, 18.5–24.9	70,990(73.1)	65,772(73.3)	5218(71.7)
Overweight and obese, ≥25	10,387(10.7)	9551(10.6)	836(11.5)
GWG^b^			
Low	17,246(18.2)	15,917(18.1)	1329(19.1)
Appropriate	44,904(47.3)	41,932(47.6)	2972(42.8)
High	32,887(34.6)	30,238(34.3)	2649(38.1)
Educational level			
High school or less	34,503(36.4)	32,419(36.2)	2084(38.6)
Vocational school/College	39,886(42.0)	37,680(42.1)	2206(40.8)
University or higher	20,509(21.6)	19,395(21.7)	1114(20.6)
Occupation during pregnancy^c^			
Non-employed	31,192(34.1)	29,422(34.4)	1770(29.4)
Low physical job	36,271(39.6)	33,884(39.6)	2387(39.6)
Moderate physical job	19,862(21.7)	18,254(21.3)	1608(26.7)
High physical job	4232(4.6)	3971(4.6)	261(4.3)
Smoking during pregnancy			
Yes	4362(4.6)	4052(4.6)	310(5.6)
No	90,285(95.4)	85,049(95.5)	5236(94.4)
Drinking alcohol during pregnancy			
Yes	2672(2.8)	2521(2.8)	151(2.8)
No	91,982(97.2)	86,679(97.2)	5303(97.2)
Chronic diseases			
Yes^d^	2678(2.8)	2453(2.8)	225(3.2)
No	92,702(97.2)	85,858(97.2)	6844(96.8)
Newborn weight for age			
SGA	7471(7.7)	6767(7.5)	704(9.6)
AGA	79,958(82.3)	74,040(82.4)	5918(80.5)
LGA	9744(10.0)	9010(10.0)	734(10.0)

*AGA* appropriate-for-gestational-age, *BMI* body mass index, *GWG* gestational weight gain, *LGA* large-for-gestational-age, *SD* standard deviation, *SGA* small-for-gestational-age

^a^Note that the numbers in the columns do not add up to total numbers

^b^GWG was stratified by applying the appropriate GWG ranges of 9 to12 kg, 7 to 12 kg, and 5 to 7 kg for underweight, normal-weight, and overweight and obese pregnant women, respectively

^c^Categorised as ‘unemployed’ (including students and women not classifiable by occupation), ‘low physical job’ (e.g. managers, engineers, and clerks), ‘moderate physical job’ (e.g. sales, services, and security workers), and ‘high physical job’ (e.g. manufacturing process workers, farmers, construction workers, carrying workers, and cleaners)

^d^diabetes, hypertension, heart disease, or kidney disease

## Discussion

The relationship between maternal energy intake and birth weight is often overlooked because it is mediated by GWG. In this Japanese cohort of non-anomalous singleton pregnancies, energy intake during pregnancy was positively associated with birth weight. Specifically, we observed absolute mean birth weight increases of 19 g and 30 g when moving from low energy intake to moderate and high energy intakes, respectively. This association persisted after adjusting for pre-pregnancy BMI, smoking during pregnancy, gestational age, and other established confounding factors. Notably, a significant portion of the association was mediated by GWG. Previous studies [12–14] have not investigated this relationship, and GWG may have obscured the associations between energy intake and fetal growth and birth weight in those studies. Moreover, the characteristics of participants and the method of handling energy intake values may partly explain the differences in findings between studies. Factors such as pre- and peri-conceptional nutritional status of participating women, different dietary patterns and energy composition, and the exclusion or inclusion of preterm births into the study might determine the observed association between maternal energy intake and birth weight. Measuring energy intake as ordinal categories, as done in this study, allows effects that would have been smaller and not obvious on continuous measurement to be captured. Mediation analysis showed that GWG mediated a significant portion of the associations between energy intake and birth weight. This mediating role of GWG can be understood from the biological relationships between GWG and energy intake and birth weight. Energy intake during pregnancy is deposited in maternal and fetal tissues and is recognised as GWG [8, 9]. A GWG of 12.5 kg, for example, equates to approximately 0.9 kg of protein, 3.8 kg of fat, and 7.8 kg of water [8], and is a reflection of fetal weight together with the incremental increases in the sizes of the maternal tissues and volumes of amniotic fluid and blood [9].

During pregnancy, energy intake in both extremes increases the risk of abnormal fetal size. In our cohort, when compared with the moderate energy intake group, the low intake group had seven more women per 1000 women with an SGA birth, whereas the high intake group had eight more women per 1000 women with an LGA birth. We observed that, compared with the moderate energy intake group, the mean daily energy intakes in the low and high intake groups were 478 kcal lower and 681 kcal higher, respectively. One could argue that this difference is clinically significant. Pre- and peri-conception nutritional statuses influence the development of the placenta and mechanisms for balancing energy requirements between mother and fetus [31]. Changes in dietary habits after conception tend to be small and generally reflect pre-pregnancy intake [32]. In undernourished women with minimal energy reserves, insufficient energy intake may trigger an energy partitioning effect within the placenta. This causes a reduction in the transfer of nutrients to the fetus and subsequent restricted fetal growth. In the present study, the proportion of women with an inadequate GWG was highest in the low energy intake group. Weight gain below the recommendation may be a sign that energy intake was not meeting demand. These women might have used maternal fat and protein stores to support fetal growth, thus the lower weight gain. Also, they may compete with their fetuses for energy, thereby reducing their birth weight. In contrast, the high energy intake group had the highest proportion of women with an excessive GWG. These women might have ingested energy in excess, thus the larger newborns.

Japanese pregnant women did not comply with current guidelines on energy intake. Notably, in two-thirds of the pregnant women in our cohort, energy intake was below the recommendations. In our cohort, the estimated daily energy intake during pregnancy was 1682 kcal. In three Japanese prospective studies [13, 20, 33], it was approximately 1580–1770 kcal. In Japan, the recommended daily energy requirements for 18–29-year-old and 30–49-year-old normal-weight pregnant women with moderate physical activity levels are 2000–2400 kcal and 2050–2450 kcal, respectively [10]. A possible explanation for the observed lower energy intake is that women of childbearing age in Japan have a strong desire for small body size and some women practise self-judged dieting during pregnancy [34, 35]. Antenatal dietary counselling has been shown to have a positive effect on the nutritional intake of pregnant women, fetal growth, and newborn birth weight [36, 37]. In practice, nutritional guidance is directed mainly at preventing obesity-related complications, such as pre-eclampsia, gestational diabetes, and fetal macrosomia. In our cohort, only 10% of the women reported receiving health guidance. Studies [38, 39] have reported an increasing prevalence of underweight women of childbearing age and an increased incidence of low-birth-weight infants in Japan. In our cohort, factors, such as younger age, less education, and nulliparity, associated with poor diet quality and less favourable birth outcomes [3, 29, 30] were more prevalent in the low energy intake group. Furthermore, more women in this group conceived with extreme pre-pregnancy body weights and gained weight below the recommendations. Both SGA and LGA infants have an increased risk of adverse short- and long-term health outcomes [5, 40, 41]. We suggest that sufficient antenatal education and nutritional guidance be offered to all pregnant women. This promotes an individualised approach to ensuring optimal nutrition and an appropriate GWG, thus better pregnancy and birth outcomes.

Our findings are likely limited to Japanese women, and they may not be directly transferable to other populations around the world, which have much higher rates of pre-pregnancy overweight, obesity, and excessive weight gain. The association between energy intake and birth weight may actually be higher in other populations.

The main strengths of this study include the large sample size, which broadly represents pregnant women in Japan; the prospective design; the comprehensive information about maternal diet; and the wide range of potential confounding factors with small missingness. Since the energy expenditure of pregnant women may influence daily energy intake, fetal growth, and birth weight, we adjusted for physical activity level and occupation during pregnancy (occupational groups stratified by levels of physical activity) in our analysis.

This study also has some limitations. For example, 16% of our cohort was underweight; thus, the findings of this study may not be generalisable to the entire obstetric population. Furthermore, our analysis relied solely on dietary information collected at a single time point during pregnancy. Dietary intake could have changed according to the stage of pregnancy. Considering the various dietary changes, including those caused by the occurrence of nausea and vomiting during pregnancy, evaluating the diet at only one point does not give is not a complete dietary assessment across the entire pregnancy. However, a previous study [13] reported no significant changes in dietary intake throughout pregnancy in Japanese women. Also, the energy intake estimated using FFQs may not reflect the actual intake [42, 43]. It is also undeniable that some pregnant women may have underreported their FFQ responses. Nevertheless, the FFQ is a validated tool for grouping pregnant women according to high and low energy intake at the population level [43], and the present study analysed energy intake as ordinal categories. Moreover, we defined GWG as the last measured weight closest to birth minus the self-reported pre-pregnancy weight. Generally, self-reported weights are susceptible to underestimation, which may influence the calculated GWG. Furthermore, we defined birth size using the neonatal birth weight chart for the Japanese; thus, finding different percentages using other population charts is possible. Finally, there was a probable exclusion of women with less favourable pregnancy and birth outcomes, resulting in a possible underestimation of the risks.

## Conclusions

There is a discrepancy between studies investigating the effects of maternal energy intake on fetal growth and birth weight. The reasons could be the heterogeneity in the study cohorts and analytical methods employed. We found a positive association between energy intake during pregnancy and birth weight in a Japanese cohort of non-anomalous singleton pregnancies, where GWG mediated a significant portion of the association. Because both extremes in energy intake during pregnancy increase the risk of abnormal birth size, we suggest that nutritional guidance be offered to all pregnant women to ensure appropriate nutrition for optimal GWG and fetal growth.

## Data Availability

Data are unsuitable for public deposition because of ethical considerations and restrictions as per legal framework of Japan. It is prohibited by the Act on the Protection of Personal Information (Act No. 57 of 30 May 2003, amended on 9 September 2015) to publicly deposit data containing personal information. Ethical Guidelines for Medical and Health Research Involving Human Subjects, enforced by the Japan Ministry of Education, Culture, Sports, Science and Technology and the Ministry of Health, Labour and Welfare, also restricts the open sharing of epidemiologic data. All inquiries about access to data should be addressed Dr. Shoji F. Nakayama, JECS Programme Office, National Institute for Environmental Studies, at jecs-en@nies.go.jp.

## Abbreviations

AGA: Appropriate-for-gestational-age
BMI: Body mass index
FFQ: Food frequency questionnaires
GWG: Gestational weight gain
LGA: Large-for-gestational-age
OR: Odds ratio
SGA: Small-for-gestational-age
